# Reasons for explantation of phakic intraocular lenses and associated perioperative complications: cross-sectional explant registry analysis

**DOI:** 10.1186/s12886-021-01847-0

**Published:** 2021-02-12

**Authors:** Timur M. Yildirim, Ramin Khoramnia, Hyeck-Soo Son, Christian S. Mayer, Grzegorz Łabuz, Donald J. Munro, Gerd U. Auffarth

**Affiliations:** The David J. Apple International Laboratory for Ocular Pathology, Department of Ophthalmology, University of Heidelberg, Im Neuenheimer Feld 400, 69120 Heidelberg, Germany

**Keywords:** Complications of refractive surgery, IOL registry, IOL design, Iris-claw, ICL, Angle-supported

## Abstract

**Background:**

We discuss the safety, since their introduction, of phakic intraocular lenses (pIOLs) to correct refractive errors in healthy eyes. We investigated the reasons for pIOL explantation and the associated perioperative complications.

**Methods:**

This retrospective, cross-sectional study included 69 pIOLs, explanted at a single tertiary center between July 2005 and March 2020: 34 angle-supported (G1), 28 iris-fixated (G2) and seven posterior chamber (G3) pIOLs. Case data including the reason for explantation was taken from the patient records. Intra- and postoperative complications were evaluated for an association with the pIOL.

**Results:**

The mean duration in the eye was 10.4 (0.2–28) years. Cataractogenesis and subsequent surgery that required pIOL explantation was the reason in 42% of all cases. In 22%, cataract in combination with endothelial damage prompted explantation, with 26, 18 and 14% for G1, G2 and G3 respectively. The second most common reasons were corneal damage alone in the angle-supported group (26%), IOL subluxation in the iris-fixated group (18%), and photopic disturbance in the posterior chamber group (29%). In 68% of all explantations, the surgical course was unremarkable, while in the remaining cases perioperative complications were associated with the lens in 45.7%.

**Conclusion:**

Overall, the need for cataract surgery was the most common reason for pIOL explantation. Corneal complications were more frequent in the angle-supported pIOLs and their removal was associated with higher rates of complication compared to the other groups.

## Introduction

A variety of phakic intraocular lens (pIOL) designs have been developed for implantation to correct refractive errors in eyes with clear crystalline lenses [1]. The first record of using an IOL for this purpose was an anterior chamber IOL (ACIOL) in 1953, credited to Strampelli [2]. Choyce developed a series of nine designs intended for iridocorneal angle implantation. These lenses were made principally for cataract patients but some he implanted for the correction of ametropia in phakic eyes [3]. In the 1970s, Kelman re-designed a Choyce lens that was in turn modified by Baikoff with his ZB and ZB5M models (made by Domilens, Lyon, France) [4]. The ZB5M was later improved and made with thinner optics, larger optic diameter, flatter anterior face, and improved haptic profile to reduce angle trauma: the NuVita MA20 (Bausch & Lomb, Rochester, USA). From the outset, damage to adjacent intraocular structures, especially the corneal endothelium, was a problem with these implants [5]. The Cachet IOL (Alcon Laboratories, Fort Worth, USA) was the most recent of these angle-supported pIOLs. The company withdrew it in 2013 because of safety concerns [6].

In cataract treatment with IOL in the 1960s and 1970s, the rival procedure to angle-fixation was the implantation of iris-supported lenses. Epstein introduced his “Maltese Cross” design for pupil fixation at the same time that Cornelius Binkhorst, developed his pupil fixation lens in the early 1960s [7]. Fechner and Worst made a concave posterior surface on Binkhorst’s lens specifically for correcting phakic myopes [8]. Worst championed the procedure, at first using lenses made with a polymethylmethacrylate (PMMA) optic, the Artisan (made by Ophtec BV, Groningen, The Netherlands) and later also marketed by AMO (Advanced Medical Optics, Santa Ana, USA)/ Johnson & Johnson Vision (New Brunswick, USA), as the Verisyse. A more flexible version of the Artisan was made with a polysiloxane optic for implantation through a smaller incision: the Artiflex/(Veriflex) is on the market since 2005 [9].

Another fixation site for phakic IOLs is the ciliary sulcus. Implantation of sulcus-fixation pIOLs is associated with Federov who made the first of these lenses in the 80s from silicone material [7]. The first Implantable Collamer Lens (ICL) was implanted in 1993; and by 2000 it was approved for marketing in the United States [10, 11]. The current ICL model - commercially available since 2011 - has a central aperture aiming for a better aqueous circulation [12].

Long-term data with up to 14 years of follow-up is available for pIOLs [5, 6, 12, 13]. However, only a few studies document the reasons for explantation and there is seldom an evaluation of the intra- and postoperative course of the explantation surgery [14–17]. In most patients, pIOL removal becomes necessary as the patient ages and is considered for age-related cataract extraction with aphakic lens implantation. Therefore, intra- and post-operative complications of explantation surgery are an important aspect for evaluating these implants.

We aimed to investigate the reasons for explanting phakic intraocular lenses which had been implanted in eyes with healthy crystalline lenses and to assess the intra- and short-term postoperative course of the explantation procedure. Our data, ranging over the past 15 years, should provide a cross-sectional overview of the essential safety parameters of phakic intraocular lenses.

## Methods

We adopted a monocentric approach to ensure a standardized analysis of the clinical data. We identified 69 cases of phakic intraocular lenses explanted since 2005 where experienced surgeons performed all the explantation surgeries. Upon receipt in the laboratory, all explants were examined for morphological changes using light microscope photographs. Depending on the anatomical site of fixation we divided the IOLs into three groups; Group 1: angle-supported; Group 2: iris-fixated; Group 3: sulcus-fixated. Case data including the pIOL model, the patients age at the time of pIOL implantation, pre-explantation slit-lamp findings and endothelial cell count (ECC), date, reason and size of the main incision for explantation, intraoperative and postoperative course, was taken retrospectively from the patient records. In cases where the cornea was affected, three subcategories were made depending on the type of corneal damage: Corneal decompensation was chosen if keratoplasty was required, low endothelial cell count (ECC) if the number of cells was below 1500 cells/mm^2^ prior to explantation. In contrast to corneal decompensation and low ECC, we defined endothelial damage as abnormal corneal endothelial cell morphology, revealed by slit-lamp examination and confocal microscopy, but without corneal decompensation and with an absence of low ECC. The status of the iris tissue was assessed prior to explantation. In addition, all complications that occurred intra- and postoperatively were evaluated for an association with the pIOL. Minor postoperative changes to the iris tissue, such as small defects at the sites of the haptic enclavation, which were considered clinically irrelevant, were not considered as a complication.

The study was conducted in accordance to the tenets of the Declaration of Helsinki. It solely involves laboratory analyses of IOL explants. No additional procedures on humans or animals were performed. An ethics committee approval was therefore not required. All patients gave written informed consent on the use of their anonymized data for scientific purposes.

Data was evaluated in Excel data sheets (Excel 2011, Microsoft, Redmond, USA) and given as mean and range. Means were compared with the Kruskal-Wallis test. *P*-values below 0.05 were considered significant.

## Results

Between July 2005 and March 2020 a total of 69 phakic intraocular lenses were explanted in one center. The cohort contained 34 angle-supported (G1), 28 iris-fixated (G2) and 7 sulcus-fixated (G3) lenses. The average age of all patients at the time of pIOL implantation was 40 (18–61) years, *P* > 0.05. Explants included nine different pIOL models (Table 1). The average duration inside of the eye was 10.4 (0.2–28) years, *P* < 0.05. Group 2 had the longest time of 13.1 (0.8–28) years compared to Group 1 with 9.6 (0.9–23.4) and Group 3 with 3.2 (0.2–7.6) years. The morphological examination revealed deposits on some of the explants, but no evidence of material changes that could have caused explantation.

**Table 1:** The models of explanted phakic intraocular lenses

Number of explants	IOL Model	Manufacturer	IOL Material	Overall IOL Size (mm)	Explantation Incision size (mm)
Angle-supported (34)					
14	Phakic 6	O.I.I.	PMMA	11.5- 14.0	7.0
10	NuVita	Bausch & Lomb	PMMA	12.0- 13.5	5.5
7	Cachet	Alcon	Hydrophobic acrylic	12.5–14.0	3.0
1	I-Care	Corneal	Hydrophobic acrylic	12.0- 13.5	3.0
1	Baikoff ZB/ZB5M	Domilens	PMMA	12.5- 13.5	3.2- 3.4
1	Vivarte	Zeiss	Hydrophilic acrylic optic with PMMA haptic	12.0- 13.0	3.5
Iris-fixated (28)					
20	Artisan/ Verisyse	Ophtec	PMMA	8.5	6.5
8	Artiflex/ Veriflex	Ophtec	Polysiloxane	8.5	3.2
Posterior chamber (7)					
7	ICL	Staar Surgical	Collamer: HEMA-based polymer containing collagen	12.1- 13.7	3.2

*PMMA* Poly methyl methacrylate, *O.I.I.* Ophthalmic Innovations International, *HEMA* hydroxyethyl methacrylate

### Reasons for PIOL explantation (Table 2)

**Fig. 1:**
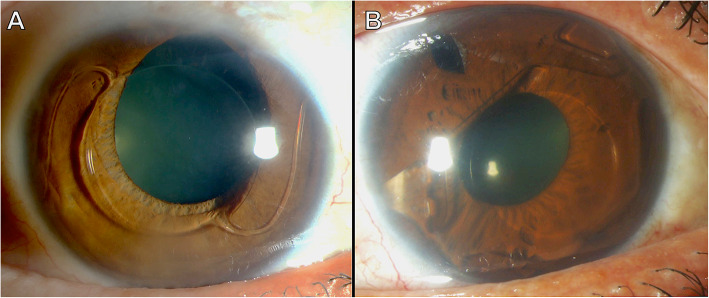
Two cases with corneal damage as the reason for explantation of the angle-supported phakic anterior chamber lens. **a:** The Safe Flex Phakic 6 IOL in a patient’s right eye caused low endothelial cell count and inferior corneal edema. Additionally, superior pupil ovalization can be seen. **b:** The decentered I-Care lens caused low endothelial cell count

**Fig. 2:**
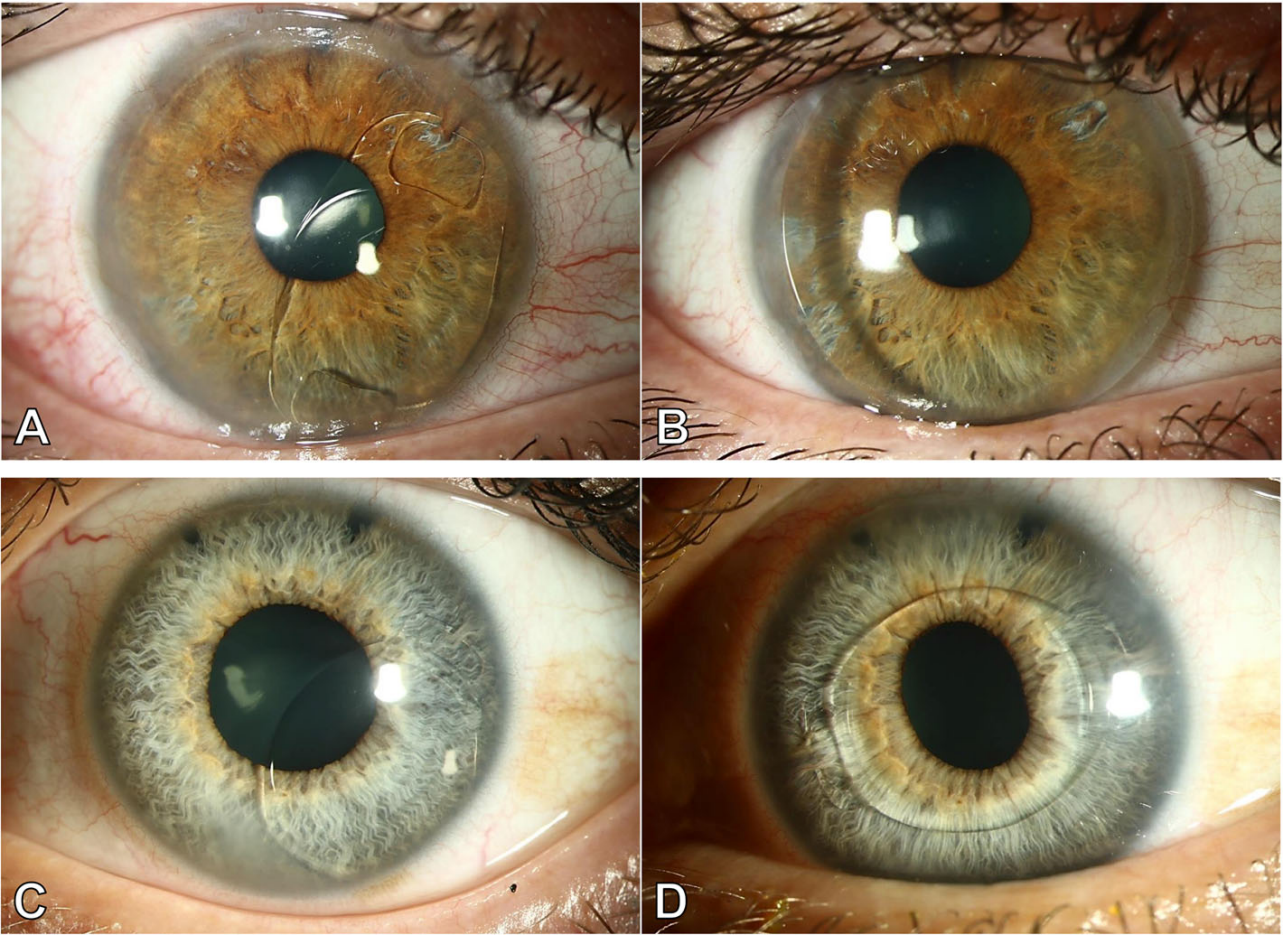
Two cases with subluxation of an iris-fixated phakic anterior chamber lens. An Artisan/Verisyse **a** and Artiflex/Veriflex **c** subluxated due to de-enclavation of the temporal **a** and nasal **c** haptic. In the first case **a + b**, decision for explantation was made and a contact lens was fitted because the endothelium was too compromised for implantation of a new lens **b**. In the second case **c + d** the lens was replaced with a new one of the same model, as one of the haptics seemed to be deformed intraoperatively but endothelial cells seemed to be uncompromised **d**. Two prophylactic iridotomies at the superior iris are visible

**Fig. 3:**
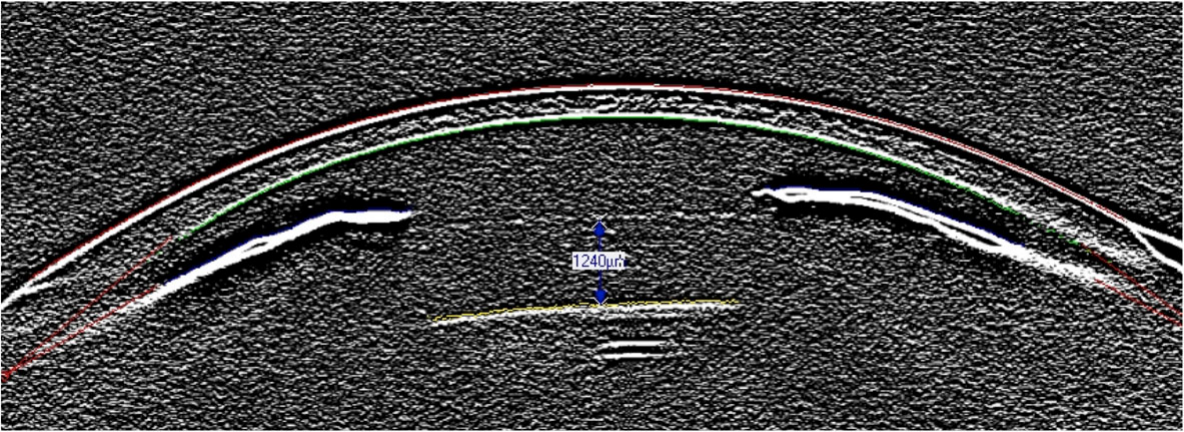
Oversized Implantable Collamer Lens. In this case a hypervault of 1240 μm is visualized using the edge filter setting of the Pentacam Scheimpflug image leading to an anterior displacement of the iris that caused intermittent raise in intraocular pressure and pigment dispersion due to iris chafing

**Fig. 4:**
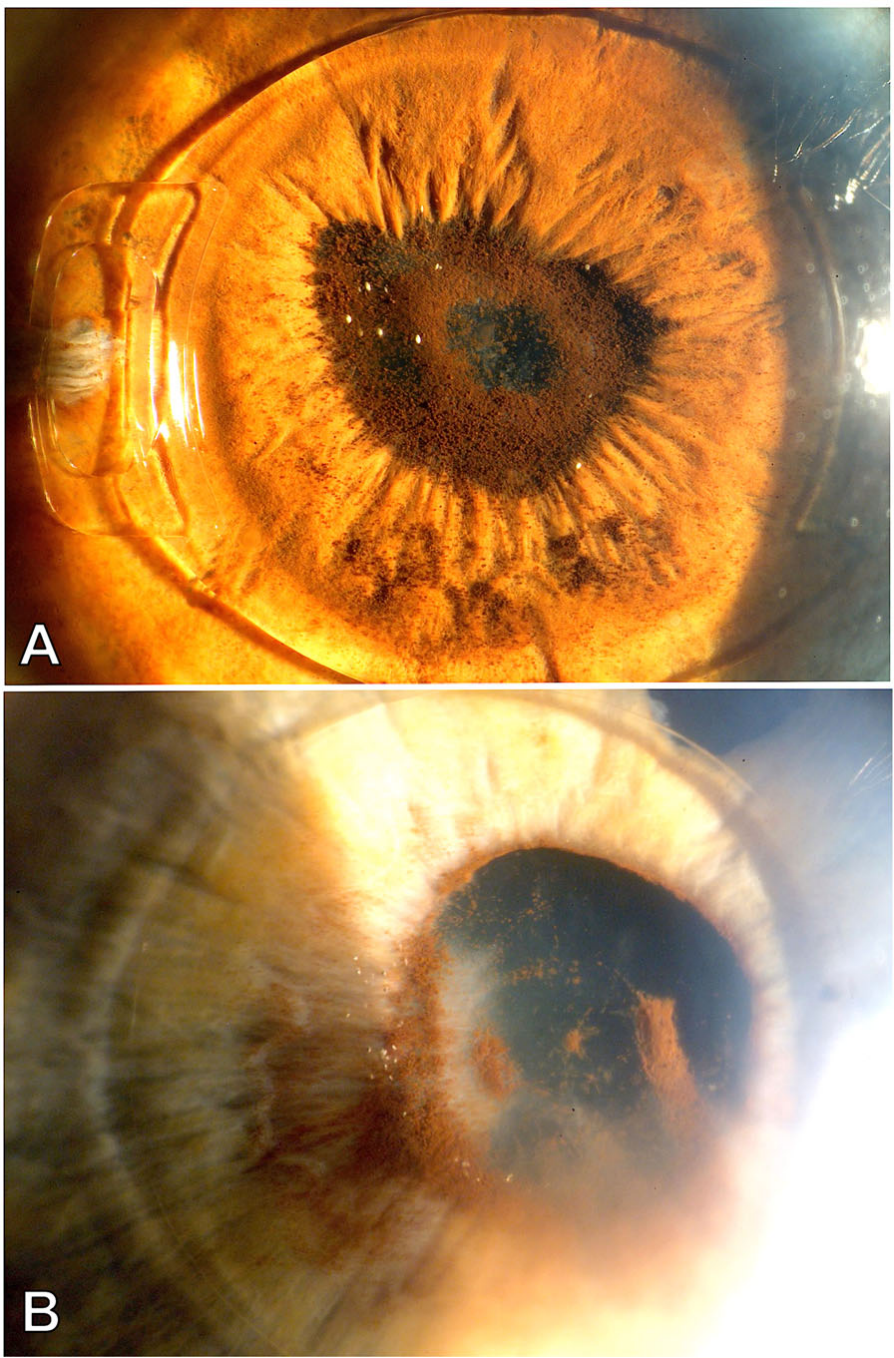
Two cases with pronounced intraocular inflammatory response due to the implant. **a**: Distinct posterior synechiae with anterior subscapular cataract. Explantation with combined cataract surgery was performed 10 months after implantation of the iris-fixated Artiflex/Veriflex IOL. **b**: Posterior synechiae with anterior subscapular cataract and incipient corneal decompensation in a case implanted with a Phakic 6 IOL. Inferior corneal edema and Descemet folds are visible. Explantation and combined cataract surgery was performed

Cataract on its own or cataract in combination with corneal damage was the most common reason for explantation, with 64% of all cases. In one third of these cases, corneal damage combined with cataract accounted for explantation, with 26.4, 18 and 14.3% in Groups 1–3, respectively. Corneal findings alone were the second most common reason for explantation in Group 1, with 26.5% cases (Fig. 1), while it was the cause in 11% of Group 2 and not present in Group 3. Eyes with low ECC as (combined) reason for explantation had an average ECC prior to explantation of 1059 (715–1476) cells/mm^2^. The second most frequent reason for pIOL explantation or exchange of iris-fixated lenses was pIOL subluxation in 18% of the cases (Fig. 2). Deformity of the de-enclaved haptic was noted intraoperatively in 60% of these lenses. In two cases from Group 3 the patient requested pIOL explantation due to unbearable persistent photopic phenomena, which resolved after pIOL explantation. In one case an oversized ICL led to anterior displacement of the iris with intermittent IOP raise and pigment dispersion (Fig. 3). In three other cases (G1: 2/34 and G2: 1/29) a pronounced intraocular inflammatory reaction led to the necessity of pIOL removal (Fig. 4).

**Table 2:** Reasons for explantation of phakic intraocular lenses

Cause	Number of cases (percentage)		
	Group 1, *N* = 34	Group 2, *N* = 28	Group 3, *N* = 7
Cataract	12 (**35.3**)	14 (**50**)	3 (**42.9**)
Cornea-related	9 (**26.5**)	3 (**11**)	
Cataract combined with corneal damage	9 (**26.5**)	5 (**18**)	1 (**14.3**)
*Type of Cataract*			
*Anterior subcapsular*	*2 (10)*		*1 (25)*
*Cortico-nuclear*	*5 (24)*	*7 (37)*	
*Nuclear*	*10 (47)*	*5 (26)*	*2 (50)*
*Posterior subcapsular*	*4 (19)*	*7 (37)*	*1 (25)*
*Type of corneal damage*^#^			
*Corneal decompensation*	*2 (11)*		
*Low ECC*	*6 (33)*	*1 (12)*	
*Endothelial damage*	*10 (56)*	*7 (88)*	*1 (100)*
Uveal tissue-related	4 (**11.8**)	6 (**21**)	
*Type of uveal-relation*			
*PIOL subluxation due to haptic de-enclavation*		*5 (83)*	
*Optic decentration due to haptic migration*	*2 (50)*		
*Chronic iridocyclitis*	*2 (50)*	*1 (17)*	
Oversizing (leading to intermitted IOP increase and pigment dispersion)			1 (**14.3**)
Photopic phenomena			2 (**28.6**)

*pIOL* phakic intraocular lens, *ECC* endothelial cell count, *IOP* intraocular pressure; ^#^ divided into three subcategories: Corneal decompensation was chosen if keratoplasty was required, low ECC if the number of cells prior to explantation was below 1500 cells/mm^2^. Endothelial damage: the presence of abnormal endothelial cell morphology but Low ECC and corneal decompensation are absent

Pupil decentration or ovalization prior to explantation was described in 58.8, 14.3 and 0% of Groups 1–3, respectively. Iris defects (other than the intended iridotomies) were only seen in Group 1, with 17.7% of the cases.

### Perioperative course

Together with pIOL explantation, cataract surgery was performed in 78.3% of the cases. The size of the main incision for pIOL explantation differed between the groups, with 4.9 (2.5–6.0), 4.8 (2.5–6.0) and 2.6 (2.5–3.0) mm for Groups 1–3, respectively, *P* < 0.05. No complications were documented during the intra- and postoperative course, in 20/34 (G1), 21/29 (G2) and 6/7 (G3) cases. In Group 1 synechiolysis was performed during explantation surgery in eight cases, in Group 2 in one case, in Group 3 no case required synechiolysis. In Group 1 the pIOL was associated with the following complications: in one case, cataract surgery had been started in an external institution and was aborted due to adhesion of the temporal haptic to peripheral iris tissue, which prevented the colleagues to explant the angle-supported lens. In two cases keratoplasty was performed for corneal decompensation due to corneal damage from the pIOL. In two cases from Group 1 and one case from Group 3, additional sutures had to be placed to prevent wound leakage. In one case of Group 2, anterior subscapular cataract was noted postoperatively, but not requiring surgery.

For other complications, association with the implant can retrospectively neither be excluded nor confirmed with certainty. Mechanical pupil dilatation for cataract surgery was required in five (G1) and six (G2) cases. Postoperative macular edema occurred in one case each in Groups 1 and 2. Three patients from Group 1 had a transiently increased intraocular pressure and one patient suffered from a prolonged intraocular inflammation which resolved completely under topical therapy. In Group 2, implantation of a capsular tension ring was performed in one case as part of the cataract surgery. In another patient of Group 2 with high myopia of − 13 and − 14 D, retinal detachment developed in both eyes, 4 and 11 months after uncomplicated combined pIOL explantation and cataract surgery.

## Discussion

The presented study provides a cross-sectional overview of the reasons for the explantation of phakic intraocular lenses over the past 15 years and describes associated intra- and postoperative complications. The distribution of phakic IOL models in this study represents the implantation pattern from about 10–20 years ago, which is different to that of the present day. Thirty-four angle-supported pIOLs are included, which are no longer available and even one very early phakic anterior chamber lens of the Baikoff type, which was implanted in a patient’s eye in 1989 and removed 23 years later during cataract surgery. Our cohort included seven Cachet IOLs - the last angle-supported pIOL model withdrawn from the market in 2013 [6]. Explantation of the Cachet was required because of low ECC (*n* = 3), optic decentration due to haptic migration (*n* = 2) and during routine cataract surgery (n = 2). Today, the ICL is frequently used for correction of ametropia [18]. Their low number in our cross-sectional study is partly due to the fact that the current popular version of the ICL has only been on the market since 2011. Furthermore, to understand the larger number of iris-fixated models in our cohort, one has to take into account historical and geographical reasons: iris-clip lenses were introduced in the 1990s and continue to be very popular in Germany.

In all three groups of our study, cataract alone (42%) or in combination with low ECC (22%) was the main reason for pIOL explantation. This total rate of 64% is consistent with previous reports [13, 14]. In a study from 2019, only including iris-fixated models, 59% of explantations were due to cataract formation [13]. An analysis from 2015 reported that 51.4% of the angle-supported, 45.8% of the iris-fixated and 65.3% of the sulcus-fixated posterior chamber lenses were explanted due to cataract formation [14]. Authors did not mention the combination of cataract and low ECC as a reason for explantation. In accordance with more recent studies, we included this combined reason as an independent reason for explantation as we found that in several cases only the combination of endothelial damage and cataract led to the decision for surgery [19].

In Group 1 corneal damage was the second most frequent reason for explantation. The natural annual endothelial cell loss is about 0.6% per year [20]. In 1999, Alió et al. presented an annual endothelial cell loss of 0.4–1.8% in 263 eyes with angle-supported pIOL of the Baikoff type over a follow-up period of 7 years after an initial cell loss of 3.8% at 3 months after implantation [5]. A more recent study from 2018 on 507 eyes treated with iris-fixated pIOLs revealed an initial cell loss of 4.6% at 6 months after implantation followed by an annual cell loss of 1.7–2.1% over a period of 10 years [19]. The threshold for pIOL explantation due to a low endothelial cell count is usually set at 1500 cells/mm^2^ [19]. This is due to the expectation that an explantation and cataract surgery can be tolerated without compromising the long-term integrity of the corneal endothelium [19]. Nevertheless, since corneal integrity does not solely depend on the absolute number of cells and endothelial cell-counting can sometimes be misleading in eyes with corneal pathologies, other factors, like the morphology of the endothelial cells in confocal microscopy, have to be taken into account when considering pIOL explantation.

A study including only iris-fixated lenses showed a similar result, endothelial cell loss was causal in one third of the 12% of lenses that required explantation within 14 years of follow-up [13]. Another study found lower values for corneal damage as a reason for explantation with 26% in the angle-supported, 29% in the iris-fixated and 4% in the posterior chamber group [14]. The higher overall rate of pIOL removal due to corneal damage of 39% in our study is mainly caused by Group 1, in which 53% of explantations were due to corneal damage, whereas the average of the other two Groups was 26%.

In Group 2, IOL subluxation was the second most common reason for explantation. Iris-clip lenses carry the risk of de-enclavation that leads to IOL subluxation [21]. This increased IOL mobility has the potential to mechanically damage the surrounding anatomical structures and, in contact with the cornea, cause a potential increase in corneal endothelial cell loss [17]. The use of incorrect instruments or surgical techniques as well as postoperative ocular trauma can promote de-enclavation [21]. In our study, one case required explantation due to a low ECC after previous direct ocular trauma had caused subsequent haptic de-enclavation and surgery for re-fixation. De-enclavation should always be critically assessed as re-fixation of the old lens might fail leading once again to a subluxation and additional endothelial damage. In a previous report we have shown, in a series of explanted subluxated iris-fixated IOLs, that irreversible deformities of the lens haptics can minimize the success of a re-enclavation [21]. In the current study, five iris-fixated IOLs had to be explanted after a prior attempt of re-enclavation. One might conclude that the better option is to directly exchange a subluxated IOL with a new one rather than attempt to reaffix it. It needs to be emphasized, however, that such an exchange should only be considered if endothelial cell density still allows the implantation of a pIOL.

In Group 3, the second most frequent reason for pIOL removal was persistent photopic disturbance. Whether the central hole in the current ICL is causal for photopic phenomena is an on-going debate. Early studies found no difference in glare or halo between the prior model and the model with the hole [22]. More recently, authors suggest that the central hole might be an additional source of dysphotopsia [23, 24]. Martínez-Plaza et al. analyzed the amount of glare in dependence of the hole’s location and found that decentration of the hole might affect patient-perceived quality of life, bothersome halogen glare, and longer recovery time from xenon glare photostress [24].

Chronic intraocular inflammation has been described in both angle-supported and iris-fixated pIOLs [25, 26]. In a comparative study, Pérez-Santonja et al. examined 30 eyes each using a laser flare cell meter at one-year and 2 years after implantation of the iris-fixated or angle-supported pIOL. While the two lens groups did not differ concerning their flare values, the values were statistically significantly higher than in a control group without pIOL [25]. In accordance with the literature, increased intraocular inflammation led to three explantations in our cohort. In one of the cases (G2), the IOL optic was made of polysiloxane. Although it is usually considered biologically less inert than PMMA, previous comparative studies between these materials have shown no differences in postoperative inflammation [26]. Hedayatfar et al. compared 16 eyes with Artisan/Verysise IOLs (PMMA optics) with 56 eyes with Artiflex/Veriflex IOLs (polysiloxane optics) - the flare values at the time points 1 week, 1 month, 3 months, 6 months and 2 years after surgery and found that this did not differ between the two groups at any time point of the follow-up period [26]. Mechanical irritation seems to be responsible for the inflammatory reaction in pIOLs [25, 26].

Besides the reasons for explantation, we also focused on evaluation of the intra- and postoperative course of pIOL explantation surgery, since this complements the safety profile for each lens design. The size of the main incision required for pIOL explantation chiefly depends on the lens material. Where the optic was made of a rigid PMMA, it cannot be explanted through the small incision that is standard in modern cataract surgery, approximately 2.2 to 2.8 mm, The explantation of a pIOL with a rigid PMMA optic requires an incision of up to 6.5 mm (Table 1) [27]. Even though creation of a corneoscleral tunnel minimizes its risk, wound-related complications might be higher in these cases. Softer optic materials, such as those made of polysiloxane or hydrophilic acrylate could be advantageous, as they allow explantation through smaller incisions.

In Group 1, synechiolysis had to be performed more often during explantation surgery than in Group 2; while no synechiolysis was required in Group 3. Intraocular adhesions requiring additional manipulation carry the risk of subsequent complications [28]. Sammouh et al. observed haptic migration through the iris tissue in 23% of 35 eyes treated with angle-supported (Phakic 6) pIOLs [29].

In a previous study on explanted phakic IOLs, seven of 240 cases required combined keratoplasty at pIOL explantation. However, the authors did not differentiate between the types of phakic lens models [14]. In the presented study, in two cases from Group 1, penetrating keratoplasty had to be performed due to decompensated cornea.

For the remaining intra- and postoperative complications, an association with the pIOL could retrospectively neither be excluded nor confirmed. However, the transient postoperative rise in intraocular pressure and macular edema tend to suggest a connection with the surgical trauma more than with the presence of a pIOL [30, 31]. Likewise, the retinal detachments following uncomplicated combined pIOL explantations and cataract surgery seen in a case from Group 2, seem to be more likely connected to the predisposing high myopia, cataract surgery and the surgical trauma itself [32]. We acknowledge our study has the limitations that it is a descriptive study, and due to its design further statistical analysis was not feasible.

## Conclusion

This study provides an overview of the reasons for pIOL explantation and its associated complications. While corneal damage was the second most frequent reason in Group 1 (no longer available), other causes, like IOL subluxation and photopic phenomena appear to be of higher relevance in today’s phakic IOLs. The explantation procedure of angle-supported lenses was associated with a higher rate of perioperative complications compared to the pIOL models in current use.

## Data Availability

The datasets used and/or analyzed during the current study are available from the corresponding author on reasonable request.

## References

[1] Auffarth GU (2004). Phakic intraocular lenses. Ophthalmologe.

[2] Strampelli B (1954). Tolerance of acrylic lenses in the anterior chamber in aphakia and refraction disorders. Ann Ottalmol Clin Ocul.

[3] Pandey SK, Apple DJ (2005). Professor Peter Choyce: an early pioneer of intraocular lenses and corneal/refractive surgery. Clin Exp Ophthalmol.

[4] Baikoff G (1991). Phakic anterior chamber intraocular lenses. Int Ophthalmol Clin.

[5] Alió JL, de la Hoz F, Pérez-Santonja JJ, Ruiz-Moreno JM, Quesada JA (1999). Phakic anterior chamber lenses for the correction of myopia: a 7-year cumulative analysis of complications in 263 cases. Ophthalmology.

[6] Aerts AA, Jonker SM, Wielders LH, Berendschot TT, Doors M, De Brabander J, Nuijts RM (2015). Phakic intraocular lens: two-year results and comparison of endothelial cell loss with iris-fixated intraocular lenses. J Cataract Refract Surg.

[7] Kohnen T, Kasper T, Bühren J, Fechner PU (2004). Ten-year follow-up of a ciliary sulcus-fixated silicone phakic posterior chamber intraocular lens. J Cataract Refract Surg.

[8] Fechner PU, van der Heijde GL, Worst JG (1989). The correction of myopia by lens implantation into phakic eyes. Am J Ophthalmol.

[9] Coullet J, Guell JL, Fournie P, Grandjean H, Gaytan J, Arne JL, Malecaze F (2006). Iris-supported phakic lenses (rigid vs foldable version) for treating moderately high myopia: randomized paired eye comparison. Am J Ophthalmol.

[10] Assetto V, Benedetti S, Pesando P (1996). Collamer intraocular contact lens to correct high myopia. J Cataract Refract Surg.

[11] Brown DC, Ziémba SL, for the Collamer IOLFDASG (2001). Collamer intraocular lens: clinical results from the U.S. FDA core study. J Cataract Refract Surg.

[12] Gimbel HV, LeClair BM, Jabo B, Marzouk H (2018). Incidence of implantable Collamer lens-induced cataract. Can J Ophthalmol.

[13] Jonker SMR, Van Averbeke AAC, Berendschot T, Saelens IEY, Nuijts R (2019). Risk factors for explantation of iris-fixated phakic intraocular lenses. J Cataract Refract Surg.

[14] Alió JL, Toffaha BT, Pena-Garcia P, Sadaba LM, Barraquer RI (2015). Phakic intraocular lens explantation: causes in 240 cases. J Refract Surg (Thorofare, NJ : 1995).

[15] Pechméja J, Guinguet J, Colin J, Binder PS (2012). Severe endothelial cell loss with anterior chamber phakic intraocular lenses. J Cataract Refract Surg.

[16] Wang KJ, Zhu SQ (2010). Spontaneous dislocation of a Verisyse phakic intraocular lens with severe corneal endothelial cell loss. Eur J Ophthalmol.

[17] Coullet J, Mahieu L, Malecaze F, Fournié P, Leparmentier A, Moalic S, Arné JL (2007). Severe endothelial cell loss following uneventful angle-supported phakic intraocular lens implantation for high myopia. J Cataract Refract Surg.

[18] Scharf D, Yildirim TM, Auffarth GU, Mayer CS, Choi CY, Khoramnia R (2020). Implantation of a Phakic posterior chamber Lens in eyes with Keratoconus. Klinische Monatsblatter Augenheilkunde.

[19] Jonker SMR, Berendschot T, Ronden AE, Saelens IEY, Bauer NJC, Nuijts R (2018). Long-term endothelial cell loss in patients with artisan myopia and artisan Toric Phakic intraocular lenses: 5- and 10-year results. Ophthalmology.

[20] Bourne WM, Nelson LR, Hodge DO (1997). Central corneal endothelial cell changes over a ten-year period. Invest Ophthalmol Vis Sci.

[21] Tandogan T, Holzer MP, Choi CY, Auffarth GU, Gerten G, Khoramnia R (2016). Material Analysis of Spontaneously Subluxated Iris-Fixated Phakic Intraocular Lenses. J Refract Surg (Thorofare, NJ : 1995).

[22] Shimizu K, Kamiya K, Igarashi A, Shiratani T (2012). Intraindividual comparison of visual performance after posterior chamber phakic intraocular lens with and without a central hole implantation for moderate to high myopia. Am J Ophthalmol.

[23] Chen X, Han T, Zhao F, Miao H, Wang X, Zhou X. Evaluation of disk halo size after implantation of a Collamer Lens with a central hole (ICL V4c). J Ophthalmol. 2019:7174913. 10.1155/2019/7174913 PMC6710753 31485347

[24] Martínez-Plaza E, López-Miguel A, Fernández I, Blázquez-Arauzo F, Maldonado MJ (2019). Effect of central hole location in phakic intraocular lenses on visual function under progressive headlight glare sources. J Cataract Refract Surg.

[25] Pérez-Santonja JJ, Iradier MT, Benítez del Castillo JM, Serrano JM, Zato MA (1996). Chronic subclinical inflammation in phakic eyes with intraocular lenses to correct myopia. J Cataract Refract Surg.

[26] Hedayatfar A, Hashemi H, Asghari S, Badie N, Miraftab M (2017). Chronic subclinical inflammation after phakic intraocular lenses implantation: comparison between artisan and Artiflex models. J Curr Ophthalmol.

[27] Singh K, Misbah A, Saluja P, Singh AK (2017). Review of manual small-incision cataract surgery. Indian J Ophthalmol.

[28] Macarie SS, Macarie DM (2018). Phacoemulsification in adult patients with post-uveitis complicated cataract. Rom J Ophthalmol.

[29] Sammouh FK, Baban TA, El Ballouz HM, Warrak EL (2016). Asymptomatic haptic migration of phakic anterior chamber intraocular lens through the peripheral iridectomy. Can J Ophthalmol.

[30] Holló G, Aung T, Cantor LB, Aihara M (2020). Cystoid macula edema related to cataract surgery and topical prostaglandin analogs: mechanism, diagnosis, and management. Surv Ophthalmol.

[31] Annam K, Chen AJ, Lee IM, Paul AA, Rivera JJ, Greenberg PB (2018). Risk factors for early intraocular pressure elevation after cataract surgery in a cohort of United States veterans. Mil Med.

[32] Bechrakis NE, Dimmer A (2018). Rhegmatogenous retinal detachment : epidemiology and risk factors. Ophthalmologe.

